# Supplementary material for: Are carbon emissions trading and green financial instruments synergistic? -Comprehensive quantitative research based on content analysis
Source: PLoS One. 2024 Mar 7;19(3):e0298601. doi: 10.1371/journal.pone.0298601 (PMC10919601; doi:10.1371/journal.pone.0298601)
Supplement: S1 Data — (ZIP) [file pone.0298601.s001.zip › Data/地级市Z_F工作报告.zip!/╜¡╦╒/╤∩╓▌╩╨/└┤╫╘╣1⁄2╓┌║┼í╛┬φ┐╦╩2╛▌═°í┐_2014.docx]

各位代表：

现在，我代表扬州市人民政府向大会作工作报告，请予审议，并请市政协各位委员和其他列席人员提出意见。

2013年工作回顾

2013年，我们以党的十八大精神为指引，在中共扬州市委的领导下，团结和依靠全市人民，坚持经济建设与社会发展同步，坚持统筹兼顾与分类指导结合，坚持机制创新与方法调适并重，坚持扩大增量与优化存量并举，全力以赴稳增长、调结构、促转型、惠民生，较好地完成了市七届人大二次会议确定的目标任务。预计全年实现地区生产总值3250亿元，增长12%。财政总收入660.3亿元，公共财政预算收入259.26亿元，增长15.2%；税收收入212.75亿元，增长17.8%，占公共财政预算收入比重82.1%。预计城市居民人均可支配收入30700元，增长9.5%；农民人均纯收入14145元，增长11.5%。

1、经济运行稳中有进

注重质量效益，抓好重大项目建设。全年完成固定资产投资2000亿元。潍柴亚星等88个重大工业项目投产或开工建设。全市在建10亿元或1亿美元以上重大工业项目101项，同比增加25项；2012年竣工的21项重大工业项目新增开票销售205.8亿元、税收13.2亿元，分别拉动全市工业开票销售、税收增幅6.2个百分点和9个百分点。新竣工重大服务业项目18个，续建和新开工65个，其中新开工项目平均投资强度提高到570万元/亩。100项重大城庆工程开工73项，完成投资256亿元。

提升传统消费品质，拓展新兴市场需求。编制《城市商业网点专项规划》。市区建成4个专业批发市场。社会消费品零售总额1093亿元，增长13%。家电、汽车等消费增长较快。房地产完成投资323亿元，商品房成交521.47万平方米。推进“万村千乡市场工程”，农家店建设质量和物流配送水平进一步提升。积极发展电子商务等新业态，腾讯电商运营中心及易迅物流总部等落户扬州，金泉网实现境外上市。

培大培强骨干企业，全力扶持小微企业。分类出台支持企业发展专项政策，加大土地、信贷、用工等要素保障力度。组织银企对接活动22场，1000多户企业获批授信580多亿元。新设2亿元中小企业应急专项资金、1.5亿元小微企业信贷引导资金池。新获批省土地“点供”和独立选址项目55个。新发展私营企业15860户、个体工商户30264户；新增民营企业注册资本金实际到资503亿元，增长15.5%。畅通企业上市服务绿色通道。4家企业在省股权交易中心挂牌。

2、转型发展步伐加快

围绕技术创新和技术改造，推动工业经济质态提升。出台促进基本产业发展和企业技改的意见。全市工业增加值、开票销售、入库税收分别增长13%、13%、12%。机械装备、汽车及零部件、船舶及配套件、石油化工、新能源新光源等五大产业开票销售占全市工业70%以上。其中，汽车产业开票销售增长80%以上。上海大众仪征分公司开票销售突破200亿元。深入实施“122”技改工程，完成技改投入620亿元。高新技术产业产值占规上工业总产值比重达42.5%。建筑业完成总产值2380亿元，增长13%。获鲁班奖2项。

抓好农业规模经营，促进现代农业持续增效。开展粮食万亩高产增效创建活动，粮食产量实现“十连增”。新建高标准农田22.8万亩，新增设施农（渔）业16.1万亩。新认定省级农产品加工集中区1个，县级以上农业龙头企业销售增长25%。新增规模经营18.2万亩、工商登记家庭农场208个。新登记农民合作社409个。新增省级“五好”示范社77个。新扩建无公害蔬菜基地2500亩，改扩建百头奶牛场3个。农机、农业开发、农业科研、粮食、供销、气象在“三农”工作中发挥重要作用。

分类指导和专项支持，推进服务业提速发展。出台支持旅游、软件和信息服务、健康和养老服务发展等8项政策意见。旅游业总收入501亿元，增长15%。软件和信息服务业完成业务收入281.3亿元，增长48%。全市港口货物吞吐量9800万吨、50万标箱，分别增长11.1%、21.9%。年末金融机构存贷款余额分别增长15.9%、16.7%，汇丰银行成为进驻我市的第一家外资银行。宝应创成全国金融生态先进县。扬州国际软件园等项目相继落户。市级以上服务业集聚区达到38个。服务业增加值占地区生产总值比重41%，比上年提高1个百分点。

以人才队伍建设为重点，切实增强科技创新活力。深入推进“科教合作新长征”计划，与中关村、扬州大学签订全面合作协议。清华科技园等项目落户扬州。全社会研发投入占地区生产总值比重2.3%。专利授权量突破1万件。制定出台6个人才专项政策，新引进国家“千人计划”20人，入选省创新团队3个和省“双创计划”、“博士集聚计划”人才93名。竣工科技产业综合体120万平方米。新增国家高新技术企业85家、省级以上“三站三中心”61家。大中型企业研发机构建有率85%以上。广陵（电子信息）高新技术创业服务中心创成国家级专业孵化器。扬州成为国家创新型试点城市。

3、改革开放不断深化

突出功能性载体优化提升，稳步推进各项改革。深化市区功能区调整，蜀冈-瘦西湖风景名胜区实现扩容提升，设立空港新城和生态科技新城。有序推进市区民生保障“同城同步同标”和行政管理“同级同权同责”。平稳实施高邮乡镇区划调整。整合、组建10大国有资产管理集团（公司），加快建立以国有公司为主体的城市建设投融资体制。稳步推进高邮市、夏集镇等全国城镇发展改革试点以及江都区省农村改革试验区、小纪镇省经济发达镇行政管理体制改革试点。积极推进事业单位分类改革。深化医药卫生体制改革，启动实施城乡居民大病保险试点。

坚持“引进来”与“走出去”并举，加快发展开放型经济。成功举办“烟花三月”国际经贸旅游节、世界运河名城博览会，积极争取并承办联合国“可持续城市及可持续城镇化”高级研讨会、国际盆景大会、中法市长圆桌会议等会展活动。日韩、台湾、香港和新加坡、央企四大招商活动成效明显。全年注册外资实际到账17亿美元，新落户世界500强及跨国公司10家。宝胜与中航工业、扬农与中化集团、金方圆与德国通快等实行战略重组或合作。完成外经营业额5.3亿美元。实现进出口总额95亿美元，其中出口75亿美元。

围绕产业集聚、特色发展，促进各类园区提档升级。实施省级以上开发区个性化考核，出台促进工业集中区整合提升特色发展的意见。全市园区业务总收入、公共财政预算收入均增长25%以上。7家省级以上开发区分别与上海、苏南等地园区签订共建协议。扬州开发区全国首批循环经济教育示范基地挂牌，扬州高新区成为省首批文化科技产业园区。仪征、邗江分别跻身省汽车产业基地（乘用车）和汽车零部件产业基地。

4、城乡建设再上台阶

争取到省“一市一试点”的专项扶持，融合发展实现新突破。省委省政府出台了《关于推进扬州跨江融合发展综合改革试点的意见》。实施《沿江地区融合发展行动计划》，组织编制江广融合地区概念规划、城市设计及“七河八岛”地区空间管制规划，逐步构建“一核多组团”城市空间布局。推进沿江沿河地区协调发展，争取到省委省政府扶持高邮宝应地区享受苏北政策。推动《宁镇扬同城化发展规划》的制定与实施。

推进全市域路网规划与建设，重大基础设施建设取得新进展。连淮扬镇铁路项目建议书获国家发改委批准。编制完成扬州城市轨道线网规划。宁启铁路复线及电气化改造稳步推进。文昌路西延、新万福路等开工建设。瘦西湖隧道盾构全线贯通，西部交通客运枢纽主体封顶。新淮江公路江都段、328国道连接线等全线通车。建成长江万吨级以上泊位5个。淮河入江水道整治完成切滩1050万方。南水北调东线一期工程扬州段率先建成通水。

注重品质提升，进一步完善城市基本功能。制定并实施城市地下空间开发利用管理办法和意见。加快建设“清水活水、不淹不涝”城市，整治城区积水路段14处，完成城市东部水系沟通活化工程和二道河等3条河道生态清淤。深入推进“三拆三整治”，拆迁拆违400万平方米。新（改）建道路40条、桥梁9座。宋夹城体育休闲公园基本建成。启动实施生活垃圾焚烧厂二期、建筑垃圾综合利用处理厂、餐厨废弃物处理厂和赵庄垃圾填埋场增高扩容工程。继续推进第一水厂提标扩建、六圩污水处理厂三期等工程建设。

突出治水和绿化，不断加强生态文明建设。全市完成造林7.06万亩，新创省级绿化示范村119个。集中式饮用水源地水质达标率100%，重点流域水污染防治考核断面水质达Ⅲ类标准。村庄环境整治任务全面完成，100个村庄达到省三星级“康居乡村”标准。疏浚县乡河道210条、村庄河塘2360条（面）。重点实施130项节能工程和147项减排工程，节能减排完成省定目标。获批国家水生态文明建设试点城市。国家生态市创建通过环保部公示。

5、社会建设和谐稳定

大力促进就业创业，健全完善社会保障。全年新增城镇就业6.7万人、转移农村劳动力3.9万人。城镇登记失业率2.41%。开展创业培训1.2万人，建设创业孵化基地10个。通过创新金融产品、鼓励民间投资、加快农村土地流转等多种方式推进城乡居民财产性、经营性收入增长。连续九年上调企业退休人员基本养老金。为全市8.7万低保对象发放补助1.2亿元、1.7万五保对象落实供养资金6266万元。城乡低保标准增幅达20%。城乡基本养老保险、基本医疗保险覆盖率稳定在98%以上。市区被征地农民社会养老保险工作稳步推进。

围绕百姓需求，扎实做好各项民生工作。保持物价基本稳定，居民消费价格指数102.2。全市改造更新区域供水入户管网552公里。新（改）建农村公路190公里、农村危桥170座。市区新增保障住房5617套（户）。全市住房公积金扩面7.7万人。深入推进市区“八老”改造，整治老小区101万平方米、公房解危4.75万平方米、改造“城中村”15个。出台《关于进一步加强市区物业管理工作的意见》。新（改）建住宅小区停车位3000个。对11个农贸市场进行局部改造。市区公交分担率20.6%，新辟优化公交线路13条，建成文昌路公交快线。新建120个农村社区居家养老服务中心（站）、92个城市社区日间照料中心。安置残疾人就业1510人次。全市30%以上低收入农户实现脱贫。

统筹发展社会事业，不断提升公共服务水平。新（改）建公办幼儿园10所。全面完成中小学校舍安全工程三年任务。高中教育质量稳步提高。2所职教学校接受首批国家教育改革发展示范学校创建验收。邗江通过义务教育基本均衡发展国家级评估。建成全国示范社区卫生服务中心1家、省示范服务中心22家。实施11类43项基本公共卫生服务项目。市第三人民医院获批三级医院。村级文化广场实现全覆盖，新建市图书馆分馆3个。文化惠民活动广泛开展。木偶剧《胡桃夹子》等入选中国艺术节优秀展演剧（节）目。大运河申遗工作通过联合国教科文组织专家考察。隋炀帝墓考古发掘入选2013年全国考古六大新发现。扬州鉴真国际半程马拉松赛蝉联国际田联“金标赛事”。城市社区“十分钟体育健身圈”建成率超过80%。《扬州市志1988-2005》通过终审。城市文明程度指数测评名列全国第四。人口计生、科普、台湾事务、侨务、外事和工会、青年、妇女、儿童、老龄、慈善等事业取得新进步。

夯实基层基础工作，保障社会稳定有序。推进“四位一体”社区治理模式和网格管理服务体系。完成全市村（居）委会换届选举工作。突出“四项排查”，深化社会稳定风险评估，完善社会治安立体防控体系，全市矛盾纠纷调处成功率达98.6%，公众安全感连续11年保持94%以上。实施食品药品专项整治，江都、仪征创成省级药品安全示范县。突出对重点领域安全监管，加强安全生产的制度建设与执行，事故数、死亡数分别下降1.7%、2.7%。应急管理基础工作进一步加强。创成全国首批法治城市创建活动先进市。国防动员和后备力量建设取得新成效。双拥、民族、宗教、民防、地震、仲裁等工作扎实开展。

6、政府自身建设不断加强

强化效能提升，能力作风持续改进。继续开展“三下三联三交”活动。出台市《行政效能问责暂行办法》。建立涉企检查、处罚报备制度。实施“减章、减时、减负、提效”行动，取消和调整市级行政审批事项59项，向广陵区下放行政权力55项、派驻行使权力15项。完善重大项目审批“绿色通道”制度。推进基本建设项目并联审批改革，流程办理时间缩短1/3以上。12345政府服务热线和“寄语市长”满意率85%以上。

坚持依法执政，行政行为规范有序。主动接受市人大及其常委会的法律监督、工作监督和市政协的民主监督，广泛听取各民主党派、工商联、无党派人士和社会各界意见。全年办理市人大代表议案、建议252件；政协提案397件，其中市长领办5件。加强政府信息公开和新闻发布工作。在全省率先实行行政执法情况通报制度。全面清理行政执法主体和规范性文件。建立市行政复议委员会，全年办理行政复议案件222件。推行城建项目“强化设计导则、方案优化组合、效果群众评估、质量延伸审计”制度。

注重制度建设，勤政廉政得到加强。坚决贯彻落实中央“八项规定”和《党政机关厉行节约反对浪费条例》。强化财政预算执行管理和政府专项资金绩效管理。组织实施在“三直接”领域对十大环节的监管。市公共资源交易中心挂牌运行。执行好“三公开三报告”制度，12个单位参加电视直播接受群众评议。强化效能监察和审计监督，开展工程建设等领域突出问题专项整治。继续实施好纪检监察组派驻督查亿元以上重大项目。

各位代表！以上成绩的取得，是中共扬州市委正确领导的结果；是市人大、市政协有效监督、大力支持的结果；是全市人民攻坚克难、共同奋斗的结果。在此，我代表扬州市人民政府，向全市人民，向人大代表、政协委员，向各民主党派、工商联、无党派人士、各人民团体，向驻扬部队、驻扬单位，向所有关心、支持、参与扬州建设和发展的同志们、朋友们，表示崇高的敬意和衷心的感谢！

同时，我们也清醒地看到经济社会发展中还存在一些深层次矛盾和问题，主要是：产业规模不大和企业核心竞争力不强的问题还很突出；区域协调发展与城乡统筹发展还有大量工作要做；城市发展的动力与城市治理的能力有待进一步提升；发展方式转变与生态文明建设的任务还很艰巨；转变政府职能与提升民生工作的社会满意度仍需加强，等等。对此我们必须高度重视，并采取切实措施认真加以解决。

2014年工作安排

2014年是全面贯彻落实党的十八届三中全会精神、实施跨江融合发展综合改革试点的开局之年，也是全力推进“十二五”规划、迎接扬州建城2500周年的关键之年。做好今年工作，既有改革活力增强、转型步伐加快、融合发展提速等有利因素，也面临着经济形势复杂多变、资源要素紧缩等诸多挑战，需要我们进一步以创新的思维、改革的理念，攻坚克难，求真务实，协力奋斗。

2014年政府工作的总体要求是：全面贯彻落实党的十八大、

十八届三中全会、中央经济工作会议和省委十二届六次全会、省经济工作会议精神，按照市委六届六次全会的部署和要求，坚持“稳中求进、进字当先，改革创新、融合发展”的总基调，以跨江融合发展综合改革试点为总抓手总平台，更大力度推进改革开放、创新转型；更大力度实施融合发展、城乡统筹；更大力度建设生态文明、秀美扬州；更大力度抓好社会建设、民生改善，努力实现经济社会持续快速健康发展，切实推动名城建设再上新台阶。

2014年全市经济社会发展的主要预期目标为：地区生产总值增长10%，公共财政预算收入增长10%，社会消费品零售总额增长13%，固定资产投资增长18%，全社会研发投入占地区生产总值比重达到2.35%，城乡居民收入增长与经济增长同步，居民消费价格指数低于全省平均水平，城镇登记失业率控制在3.5%以内，节能减排完成省下达任务，严守占国土面积20.11%的生态红线区域。

重点抓好以下九个方面工作：

1、突出深化改革，全力推进跨江融合发展综合改革试点

按照市委“改革全面深化年”的部署，抢抓省委省政府赋予我市的政策机遇，把改革作为今年政府工作的首要任务，全力推进市场开放、改革攻坚、体制“破冰”。

深化经济体制改革。打造公平开放透明的市场环境。推进工商注册便利化。改善投资管理方式，开放投资领域，培育和吸引多元化投资或运营主体。建立健全社会征信体系。促进金融创新发展。培育壮大地方法人金融机构，加快金融集聚区和科技金融合作创新示范区建设。多渠道推动股权、债券融资。推动国有企业完善现代企业制度，积极发展混合所有制经济。推进城投、交投、水务、现代金融等国有投融资公司优化资产重组，发挥国有资本在城市建设和公共服务中的骨干作用。优化旅游发展体制，整合旅游资源，提高市场化运作水平。做好第三次经济普查工作。

深化行政体制改革。推进行政审批制度改革，全面清理、规范各类行政权力，向社会集中公布行政权力目录清单、涉企收费清单、行政审批并联操作流程图，形成行政权力阳光透明高效运行体系。推进与行政审批相关的中介机构规范化市场化改革。实行全面规范、公开的预算制度。严格政府债务控制和管理，提高资金使用效率并建立健全偿债机制。推进政府向社会组织购买公共服务，通过引入竞争机制，提高服务水平。

深化城市治理体制改革。推进各区、各功能区加快形成发展定位清晰、功能明确、权责一致、治理有效的治理体系。进一步深化市区三区管理体制“同级同权同责”。加快建立市、区两级财力与事权责任相匹配的新型财政关系和市区各功能区投入收益平衡机制。对涉及城市全局的主干道路和主要河道、地上线网和地下管网由市统筹规划、统一监管。推进城管综合执法改革试点。注重城市运行安全，消除城市安全隐患。

深化社会事业领域改革。加快事业单位分类改革。推进文

化体制机制创新，鼓励社会力量、社会资本参与公共文化服务体系建设。实施教育领域综合改革。优化整合市区各项教育资产，理顺市区教育管理体制。推行校长教师交流轮岗，进一步促进基础教育优质均衡发展，宝应、高邮、江都通过义务教育基本均衡发展国家级评估。加快职业教育资源整合，组建市职教集团，进一步提升职业教育与企业需求的契合度。筹建旅游类职业技术学院。深化医药卫生体制改革。加快县级公立医院综合改革。扩大基层基本药物制度覆盖面，公办基层医疗卫生机构基本药物配备使用率达100%。组建新的食品药品安全监管机构，建立严格的覆盖全过程的监管制度。

2、突出创新转型，切实提高发展的质量和效益

进一步集聚创新资源，转变发展模式，“创”出核心竞争力，“转”出现实生产力，打造发展新能级。

提升发展先进制造业。放大我市省级整车生产基地的品牌效应，做大做优零部件产业链和生产规模以及汽车后市场，重点推动新能源汽车关键技术研发及示范应用。提升机械产业自动化成套化水平，加快建设五大机械装备制造基地和六个特色机械产业集聚区。优化整合船舶产业，积极培育船舶配套和海洋工程装备制造业。促进化工园区链式发展、绿色发展。推动光伏产品市场开发和LED产业终端应用产品的开发，加快光伏电站建设和分布式光伏发电项目实施。采取专项措施，引导企

业运用高新技术改造提升传统产业，做好相关产业过剩产能的

压缩性调整。全年工业增加值增长11%以上。

全力发展现代服务业。全面落实发展服务业各个专项政策，加快转型升级，服务业增加值占地区生产总值比重达到42.5%。树立“城市即旅游”的理念，大力发展文化旅游、商务旅游、会展旅游，加快旅游度假区等载体建设，推进市县联动，加大城市旅游营销力度，旅游业增加值占地区生产总值达到7%左右。围绕推进新兴软件名城建设，重点打造广陵、邗江和市开发区三大板块，软件和信息服务业实现业务收入420亿元，增长50%。培育壮大电子商务业，推动腾讯等电商龙头企业做大规模，引进国内电商百强企业5家以上。通过打造产业基地、建设人才队伍、组建发展基金等措施，全力扶持非遗文化产业发展。文化产业增加值占地区生产总值比重达到4.8%。加快培植健康和养老服务产业，积极发展亲子教育、体育健身、中医药健康、养老服务等全生命周期健康服务。推动传统商贸、现代物流业加快发展，培育年交易额过百亿元批发市场2个、年营业收入超10亿元物流集聚区1家。加快空港新城建设，集聚培育空港现代物流业、与航空关联的制造业。推进扬州港“亿吨大港、百万标箱”建设，理顺管理体制和优化运行机制。

积极实施创新驱动。推进国家创新型试点城市建设。推动与中科院、中关村、扬州大学项目化合作取得实质性进展。推进市产业技术研究院建设。组织实施100项产业创新链关键共性技术的研发和应用。深入实施“品牌、标准、专利、上市”四大战略，创省级以上品牌20件，启动知识产权强市规划建设，制（修）定国家或行业技术标准8项以上，争取授权专利1万件，力争新增上市企业2家以上，支持企业进入“新三板”等场外交易市场挂牌。全面提升8个国家级高新技术特色产业基地建设水平，加快推进省级科技服务示范区建设。新建产业特色鲜明的科技产业综合体50万平方米，提高综合利用效率。落实好系列人才政策，新引进高层次领军人才80名、产业急需专业技术人才1000名。

切实优化投资消费结构。进一步改善投资结构，优先发展高端装备制造、节能环保、农田水利等国家鼓励类项目，扩大现代服务业投资规模，实现工业投资占全部固定资产投资比重60%以上。适应消费结构升级和变化趋势，继续巩固批零住餐等传统消费，稳步推进低碳住宅、新能源汽车等绿色消费，积极扩大节能产品、信息消费等政策鼓励类消费，大力拓展便利服务、文化休闲和具有主题特色的城市综合体等品质消费。畅通商品流通环节，加强产品质量监管，营造安全消费环境。

3、突出服务企业，促进经济平稳健康发展

进一步强化“企业强则扬州强”的意识，着力解决企业面临的突出问题，净化发展环境，积蓄发展后劲。

全力推进重大项目建设。按照“重大产业项目提升年”要求，以提升项目质量效益为核心，以投资规模、高质量就业和税收贡献、投入产出比等为重点，完善重大项目考核办法，强化项目绩效评价，推动项目建设量质并举。试行重大项目容缺受理、模拟审批、形式审查制和审批代办、领办制，进一步畅通审批绿色通道。全年新开工10亿元或1亿美元以上重大工业项目50个、重大服务业项目40个，其中基本产业项目占70%以上。

不断增强企业核心竞争力。高度重视企业家队伍建设。支持企业以改进信息化管理手段、提升智能化装备水平为重点，推进实施“122”技改工程，技改投入增长18%。实施100家创新型领军企业、100家科技型小微企业“双百企业创新发展助推计划”。鼓励中小企业“专、精、特、新”发展，加快培育一批行业专项领先的中小企业。加快企业研发机构建设，大中型企业研发经费占主营业务收入比重达1.6%。全年高新技术产业产值占比达43.5%。加大对实体经济信贷投入，制造业贷款增幅不低于全市贷款总额增幅。鼓励各类创投基金加大对科技型初创企业的投资力度，新设科技小贷公司2家、创投基金3个。

大力发展开放型经济。继续办好“烟花三月”国际经贸旅游节、世界运河名城博览会，积极争办国际会展、大型企业董事会、年会等。扩大开放与合作，新引进世界500强及跨国公司投资项目8个、央企合作项目30个、知名企业研发中心和高校院所创新中心20家。注册外资实际到账增长10%。优化出口结构，外贸出口增长5%。支持企业参与境外项目投资开发，外经营业额增长15%。加快园区转型升级，推动江都和仪征经济开发区争创国家级开发区，扬州高新区争创国家级高新区，扬州出口加工区争创综合保税区。加快省级以上开发园区与上海张江高新区、苏南国家级开发区合作共建。力争实现扬州泰州机场一类口岸开放。推进特色园区的国际交流与合作。

加快释放民营经济发展活力。出台支持全民创业加快民营经济发展的意见。坚持“非禁即入”，努力消除民营资本进入的各种隐性壁垒，制定民营企业进入特许经营领域的具体办法。加大对全民创业特别是初次创业者的支持力度。制定允许企业以知识产权、土地使用权、股权、债权等作价出资的办法。推行轻微违章“首违不罚”和“容期整改”制。设立企业“安静生产日”。切实帮助小微企业和个体工商户解决融资难融资贵等问题，年内新增个转企1200家以上。新增民营企业注册资本金实际到资530亿元。新增列统企业500家以上。

4、突出“三农”提升，加快推进城镇化和城乡一体化建设

坚持以工业反哺农业、城镇化带动农村、产业化致富农民，走城乡互补、工农互惠的发展之路。

深入推进农业现代化。稳定粮食生产。做好危旧粮仓改造。继续推进6个中央财政小型农田水利重点县和200万亩高标准基本农田示范项目建设。加快现代农业产业园区建设，新增高标准农田、设施农（渔）业各15万亩。高效设施农业保险覆盖面40%。推进农业“接二连三”，支持农产品深加工、农超对接。新培育亿元以上农产品加工龙头企业20家。新增“三品”认证120个。加快构建新型农业经营体系，培育新型农业经营主体。全年新增土地集中型适度规模经营10万亩、省“五好”示范农民专业合作社30个、市级示范家庭农场10个。

推进城镇化健康发展。坚持以人为本和生态优先，进一步优化城镇规划布局和强化土地集约利用。加强城乡服务体系一体化建设和社会保障转换对接工作。有序推进农业转移人口市民化进程。每个县（市）区选择1-2个各具特色的城镇化综合改革试点镇。推进农村土地经营承包权确权登记和颁证工作。建立农村综合产权交易市场。加快江都农村改革试验区建设，争取扬州和有条件的县（市）区进入省城乡发展一体化试点。创新投融资模式，引进民间资本参与新型城镇化建设。新（改）建农村公路180公里、农村危桥88座。全市镇村公交开通率达到60%。

做大做强县域经济。依靠科技与人才，发挥政策拉动作用，进一步做大产业规模，促进高邮、宝应地区提速提质发展。加快省级开发区和工业集中区提档升级集聚发展，继续按重点发展型、优化发展型、控制发展型对集中区进行分类指导，支持高标准厂房和集中区的综合服务功能建设。深入推进村级“四有一责”建设，全市村级集体经营性收入增长10%以上。严格控制村镇债务。强化交通对县域经济的支撑带动作用，建成新淮江公路、邮仪公路，加快推进文昌路西延工程，启动环邵伯湖大道规划建设。

5、突出名城建设，不断提升城市功能和品质

围绕打造“宜居宜游宜创、产业就业置业”城市和“一核多组团”格局，提高组织程度，强化建管质量，全力完成“重大城建项目决战年”各项任务。

加快推进重大基础设施建设。积极策应上海自贸区、长三角区域一体化、长江经济带和宁镇扬同城化、苏南现代化示范区建设，加快构建与长三角核心区优联快通的交通网络。全面完成宁启铁路扬州段复线及电气化改造。推动连淮扬镇铁路开工、扬州高铁站枢纽规划和开工建设扬宿高速，推进京沪高铁宁扬联络线、宁（仪）扬城际轨道、京沪高速扩容和南延、沪陕高速江广段和西北绕城高速公路扩容等的前期工作。改扩建扬州港区3号泊位码头，续建江都港区3-5号泊位，建成仪征港区2个5万吨级液体化工泊位，建设高邮运东船闸扩容工程。加快实施淮河入江水道整治工程。推进扬州配电网示范区项目建设。

进一步做优做美中心城市。加快东部生态科技新城和广陵新城、中部瘦西湖景区和古城、西区新城、南部临港新城板块建设，进一步优化城市发展格局。全力实施100项重大城庆工程，加快推进交通银行总部第三基地、科技馆等项目建设，建成体育公园体育场。优化古城保护和利用，继续做好南河下民居改客栈工作。推进瘦西湖水系沟通和瘦西湖路景观提升，完成“双峰云栈”复建和旅游服务中心改建。打造“双宁”文化核心区，推动“双东”历史街区争创国家5A级景区。执行市人大关于加强“七河八岛”区域生态环境保护的决议，以实施农业结构调整为切入点，落实好“四控一禁”。全面建成瘦西湖隧道、西部客运枢纽，加快推进新万福路建设，抓紧启动金湾路、邗江路南延、江都路北延等重点项目，超前谋划城市快速环路建设。实施“五路一环一河”、城区高速道口等重要路段的环境改造和区域的景观美化。进一步提升房地产项目的功能和品质。

不断提升城市精细化管理水平。推进数字化城管向县市延伸，完成江都数字化城管区级平台建设，年内实现市区全覆盖。继续推进城市环境综合整治，实施新一轮“三拆三整治”。新建垃圾中转站2座、新（改）建公共厕所99座。提高环卫作业市场化、机械化水平，“五路一边”环卫机械化作业率达100%，其他道路机械化作业率40%以上。优化城市核心区交通网络，继续推进文昌阁地区中环疏解工程。提高交通组织管理能力，市区道路、桥梁等城建项目100%开展交通影响评估，切实降低项目施工对市民出行的影响。

6、突出生态文明，努力打造秀美扬州

实行最严格的生态环境保护制度，大力实施“绿杨城郭”、“碧水蓝天”工程，让生态宜居成为扬州第一资源、第一品牌。

强化生态文明制度建设。以更高标准编制全市生态文明建设规划。建立健全自然资源资产产权制度。严格实施生态红线保护规划，建立健全区域资源有偿使用制度和重点生态功能区生态补偿机制。构建循环经济工作推进体系。建立生态文明建设多元化投入机制，加大公共财政对生态环保投入力度。继续实施土地优供、限供和不供的差别化供应机制，盘活存量土地，确保土地资源的集约利用和土壤质量的改善保护。

抓好水环境和大气治理。治城先治水。大力推进全国水生态文明试点城市建设。启动市区“清水活水”三年综合整治工程，今年先期实施市区新城河等5条河道整治。加快古运河和小秦淮河环境提升、城市西部调水引流工程，年内实现城区主干河道活水全覆盖。做好新建住宅小区雨污分流配套工程。用两年时间完成全市农村河塘整治，全年轮浚县乡骨干河道230条、村庄河塘6000条（面）。全面落实河道管理“河长制”。继续实施汤汪、六圩污水处理厂管网完善工程，铺设改造管道25公里以上。加强南水北调水源地水环境整治和监测，保障供水安全。加大对机动车尾气、燃煤锅炉、施工扬尘、餐饮油烟整治力度，按照省核定指标淘汰老旧机动车，市区淘汰燃煤锅炉80台（套），实现市区建筑工地防尘管理措施全覆盖。建立温室气体排放核算统计体系。做好省秸秆综合利用试点工作。

积极推进城乡生态建设。以“生态中心、生态廊道、生态家园、生态产业”四项工程和高邮清水潭、宝应运西、仪征枣林湾等10大生态中心建设为重点，全年造林3.5万亩，新建省级绿化示范村80个。市区新增绿地100万平方米，建成区绿化覆盖率达43%。巩固“美好城乡”建设成果，加大对农业面源污染、土壤污染防治力度。完成农村无害化户厕改造4万座。完善城乡垃圾处理体系，建成市区建筑垃圾处理厂，完成赵庄垃圾卫生填埋场增容，加快推进餐厨废弃物处理厂和生活垃圾焚烧发电厂二期工程，扩大农村生活污水、生活垃圾集中处理覆盖面。

大力实施节能减排。开展节能低碳行动，加强重点行业、重点企业、重要环节的节能减排监测管理。加快推进热电企业脱硫、脱硝等重点工程建设。推动工业园区循环化改造、工业集中区特色发展绿色发展，提升扬州环保科技产业园建设水平，扶持培育绿色环保、循环利用等产业。大力推广节能技术和产品，提高用能设备终端能效。推行合同能源管理，探索建立市场化的节能机制。市级生态工业集中区占工业集中区比重达到10%。实施节能技术改造项目120项、循环经济试点项目20个。

7、突出共建共享，不断深化民生幸福工程

坚持既发展为民又发展靠民，既尽力而为又量力而行，让群众的生活一年比一年有盼头，一年更比一年好。

切实推进居民增收。落实鼓励扶持创业就业的政策措施。全市城镇新增就业5万人，新增残疾人就业1000人，农村劳动力转移1.4万人。扬州籍高校毕业生推荐就业率100%。全年组织创业培训4000人，建立创业孵化基地10个。加强职业技能培训，中等职业教育实施订单培养、定向培养率达到65%以上，城乡劳动者职业技能培训2.25万人，努力实现劳动报酬增长与劳动生产率提高同步。发挥建筑业富民的带动作用，推进建筑业转型升级、高端发展，全年总产值增长12%。扩展投资和租赁服务，探索农村房屋、林地等资源转变为资本的办法，努力增加城乡居民财产性、经营性收入。

稳步提高社会保障水平。继续抓好社保扩面征缴工作，全市城镇职工基本养老保险、城镇职工、居民基本医疗保险和农村新型合作医疗参保率均稳定在98%以上。城乡居民养老保险参保率稳定在99%以上。全面实施城乡居民大病保险，实际支付比例不低于50%。加快社会保险市级统筹，推进社会保险“一卡通”。提升社区日间照料中心运行质态，并启动第二批建设。健全和完善城乡救助体系，设立困境儿童就学专项救助基金，继续落实城乡低保、农村五保自然增长机制，建立居民家庭经济状况核对机制。年内70%以上农村低收入农户实现脱贫。

全力办好民生实事。实施区域供水进村入户管网改造工程，改造供水支管网555.3公里。启动新一轮农贸市场提档升级三年行动计划，完成提升12个、新（迁）建3个。继续实施经济适用房货币化补贴、廉租房公租房并轨运行、保障房源社会化收储等改革。全市住房公积金扩面6万人以上。不断提高住宅物业服务覆盖率和服务水平。市区整治改造老小区55万平方米、公房解危4.5万平方米、“城中村”15个，启动实施城郊结合部环境综合整治。改造城市积水点18处。新辟调整公交线路12条，续建和新建市区公交场（站）5座。开通城市旅游公交专线，

建设公共自行车租赁系统。推进市区校车公交化。新建蔬菜生

产基地2000亩、农产品产地准出检测监控点20个。

统筹推进社会事业。健全现代公共文化服务体系，完善文化基础设施。探索文博场馆建设运营管理新机制，新（迁）建扬州八怪纪念馆、郑板桥纪念馆等。推进第一次全国可移动文物普查。全力参与并推动大运河成功申遗。开展“百场公益演出”等文化惠民活动。优化市区幼儿园、中小学规划布局，新创省优质园5所、省义务教育现代化学校60所。推进高中教育优质特色发展。异地新建特殊教育学校。以开放大学为平台整合市直行业培训资源。加强基层医疗卫生机构能力建设，开展健康促进“十大主题”行动。完善城乡医疗急救体系，新建市区急救点3个，推进苏北医院急诊中心建设。完成新农合市级信息化业务平台建设并与省级平台联通。着力提高出生人口素质。继续办好扬州鉴真国际半程马拉松赛等重点赛事。

8、突出安定和谐，创新和加强社会治理

推进以维护群众利益、增加和谐因素、增强社会活力为重点的社会治理体制建设，努力打造平安和谐扬州。

健全基层社会治理服务体制。深化推进“四位一体”社区治理模式，启动“政社互动”试点工作。解决市区全部社区服务用房。实现社区服务中心达标全覆盖。加强中介市场、新型社会组织培育。发挥市公益创投中心作用，入驻孵化社会组织35家。推动社会组织完善法人治理结构，提高社会组织开展公益服务的能力。推动专业化职业化社会工作人才队伍建设。加强流动

人口管理，推行居住证制度。

保障群众合法权益。拓宽社情民意表达渠道，关注网络民意，引导群众理性、依法表达利益诉求。健全联合接访机制，坚持领导接访下访，建立网上受理信访制度，集中化解信访积案。打造方便快捷的行政复议申请统一受理平台，健全行政复议案件审理机制。完善人民调解、行政调解、司法调解联动工作体系，健全社会矛盾大排查、大调解组织管理体系和工作运行机制，有效预防和化解社会矛盾。

维护社会和谐稳定。全面贯彻“六五”普法规划，深入推进法治文化名城建设。做好全国文明城市复评工作，放大“扬州好人”、道德模范的示范引领作用，进一步提高城市整体文明素质。健全重大决策社会稳定风险评估机制。建立隐患排查治理和安全预防控制体系。加强消防、交通运输、建筑施工和危险化学品等领域安全工作，推进安全生产标准化和安全生产责任保险。进一步提升突发事件应急反应能力。加强社会治安综合治理，创新立体化社会治安防控体系。依法打击各类违法犯罪。依法管理信息网络，建好网络社会综合防控网。健全社区矫正工作机制。进一步加强国防动员和后备力量建设，深化“七创”全国双拥模范城活动，促进军民融合深度发展。

9、突出转变职能，建设高效清廉政府

以开展群众路线教育实践活动为强大动力，以政府服务国际化和行政行为法治化为着力点，加快政府职能转变，建设人

民满意政府。

建设服务型政府。坚持群众路线、问题导向，坚持问需于民、问计于民、问效于民，提高政府服务基层、服务企业、服务群众、服务来扬旅游者的针对性和精准度。以作为求地位，以服务赢口碑，不断强化政府部门敢于担当、勇于创新、善于服务的责任意识。制定部门个性化考核办法，接受群众监督、舆论监督。实施好“减章、减时、减负、提效”行动，建立完善否决报告、首接负责和限时办结等制度，有效提升政府服务能力。

建设法治政府。切实提高运用法治思维和法治方式深化改革、推动发展的能力。自觉接受市人大的法律监督、工作监督和市政协的民主监督，认真听取各民主党派、工商联、无党派人士和社会各界意见。切实做好人大代表议案、建议和政协提案办理工作。严格执行市人大常委会关于讨论、决定重大事项的规定，完善重大事项集体决策、专家论证、群众参与制度。制定并实施与市政协协商年度工作计划。坚持依法行政，推行行政执法公开、告知、听证、说明理由等制度，切实规范行政处罚自由裁量基准。

建设阳光政府。加强政府信息公开、政府新闻发布工作。办好市长信箱、“寄语市长”栏目和12345政府服务热线。广泛推行“三直接”工作，遴选出群众最关心的新十大环节，制定出台操作规范。强化执法监察、效能监察、廉政监察，严格实行重要工作、重点项目、重大工程目标责任制和责任追究制。完善“三公开三报告”制度，将电视直播单位拓展到承担民生幸福工程和服务企业职能的所有部门。严格落实中央“八项规定”和《党政机关厉行节约反对浪费条例》，艰苦奋斗，勤俭办事，确保“三公”经费只减不增，运转性支出统一削减5%，把有限的资金和资源更多地用在社会发展和民生改善上。

各位代表！做好今年工作，任务繁重，责任重大。让我们在中共扬州市委的领导下，团结和依靠全市人民，锐意进取，开拓创新，以经济可持续发展倒逼体制机制的创新，以生态文明建设倒逼发展方式的转型，以居民收入增加倒逼更有质量效益的增长，以公共服务水平提升倒逼政府职能的转变，为推进跨江融合发展、把扬州建设成为古代文化与现代文明交相辉映的名城而努力奋斗！
